# Studies on Virulence and Extended-Spectrum *β*-Lactamase-Producing Uropathogenic Escherichia coli Isolates and Therapeutic Effect of Fosfomycin in Acute Pyelonephritis Mice

**DOI:** 10.1155/2022/8334153

**Published:** 2022-01-30

**Authors:** Lingchun Zhang, Fenfen Li, Xiaotian Li

**Affiliations:** ^1^Department of Pharmacy, Henan Provincial People's Hospital; Zhengzhou University People's Hospital, Zhengzhou 450003, China; ^2^Department of Pathophysiology, School of Basic Medical Sciences, Zhengzhou University, 100 Kexue, Zhengzhou, 450001 Henan Province, China; ^3^School of Pharmaceutical Sciences, Zhengzhou University, 100 Kexue, Zhengzhou, 450001 Henan Province, China

## Abstract

The understanding about virulence factors (VFs) and the drug resistance of uropathogenic *Escherichia coli* (UPEC) helps us understand the pathogenesis of urinary tract infections (UTIs) and make better decisions for clinical treatment. This study examined the correlation between the extended-spectrum *β*-lactamases (ESBLs) phenotype and VFs in UPEC strains. In addition, we validated the therapeutic potential of fosfomycin in acute pyelonephritis mice. From May 2017 to November 2018, 22 nonduplicate *E coli.* strains were isolated from UTI patients. PCR was utilized to detect the distribution of virulence genes. We also analyzed the ESBL phenotype in *E coli*. We further evaluated the therapeutic effect of intravenous fosfomycin treatment in the acute pyelonephritis (APN) model. All 22 UPEC strains expressed the type 1 fimbriae (*FimH*) gene and more than 50% (12/22) of strains produced ESBLs. The detection rates of the iron acquisition-associated genes *ChuT* and *IutA* were 77.3% (*n* = 17) and 50% (*n* = 11) and those of P fimbria *papA* and *papC* genes were 45% (*n* = 10) and 50% (*n* = 11), respectively. Though the VFs were closely related with pathologenicity, the relationship between VFs and ESBLs still needs further investigation. Furthermore, intravenous fosfomycin 800 mg/kg significantly reduced the bacterial load and the inflammatory infiltration in the bladder and kidney, maintaining the structural integrity of the kidney. Intravenous fosfomycin administration can be used for the treatment of acute pyelonephritis caused by highly pathogenic and drug-resistant UPEC strains.

## 1. Introduction

Urinary tract infections (UTIs) are the most common infectious disease caused by Gram-negative and Gram-positive bacteria, as well as yeasts such as *Candida* spp. [1–4]. As reported in a previous study, more than 7 million people visit physicians and nearly 50% of women in the US and European countries have UTIs at some stage of their lives [5]. Uropathogenic *Escherichia coli* (UPEC) is the causative organism and aetiologic agent responsible for 90% of all UTIs globally [1, 6]. Prolonged, indiscriminate incorrect clinical treatment with antimicrobials contributes to the generation of drug-resistant strains, especially multidrug-resistant (MDR) strains [7]. The evolution and spread of antibiotic-resistant UPEC is a worldwide health concern. Therefore, the understanding of the pathogenic mechanism of resistance must be improved to develop a treatment plan that is more suitable for UTI patients. UPEC strains express various virulence factors (VFs), which play a major role in bacterial colonization, pathogenesis, and persistence in the urinary tract [8, 9]. UPEC expresses a variety of VFs, including adherence factors (P fimbriae, S and F1C fimbriae, and type 1 fimbriae), toxins (*α*-haemolysin and cytotoxic necrotizing type 1), flagella, and iron acquisition systems [8]. Most UPEC strains express type 1 fimbriae that facilitate the adhesion and colonization of UPEC by recognizing glycoproteins on the urinary tract epithelium [10]. The adhesion of *FimH* (type 1 fimbriae) induces the reorganization of the cytoskeleton and internalization of UPEC, which may protect the bacteria from antibiotics, neutrophil influx, and shear stress [11]. In addition to type 1 fimbriae, P fimbriae (composed of six subunits: *papG, papF, papE, papK, papA,* and *papH*) recognize glycolipids (e.g., *α*-D-Gal-(1,4)-*β*-D-Gal) and interact with Toll-like receptor 4, inducing the local inflammatory response and promoting tissue destruction [10]. P fimbria also helps UPEC colonize and prevent bacterial removal in the kidney [10, 12]. The iron acquisition system facilitates iron uptake in UPEC and is critical for UPEC survival in the iron-limited urinary tract [13].

Another reason for MDR in UTI patients is that UPEC may contain extended-spectrum *β*-lactamases (ESBLs) plasmids that can clave the *β*-lactamase ring of antibiotics and induce drug-resistant EBSLs [14, 15], producing UPEC that also invade host macrophages and spread infection rapidly [14]. Although an increasing number of studies are investigating UPEC virulence, few data are available on the relationship between the EBSL-producing frequency and virulence in UPEC strains isolated from UTI patients. ZTI-01 (fosfomycin for injection) was developed for complicated UTI/acute pyelonephritis treatment in the US [7]. ZTI-01 showed good antibacterial activity towards MDR Gram-negative pathogens *in vitro* and patients complicated with UTI. However, there is a lack of sufficient evidence supporting its usage. Therefore, we conducted a study to detect the distribution of ESBLs and virulence genes in UPEC strains isolated from UTI patients. We also established an acute pyelonephritis (APN) model using the UPEC strain and evaluated the effect of the intravenous injection of fosfomycin *in vivo*.

## 2. Materials and Methods

### 2.1. Bacterial Isolates and Media

In the present study, a total of 22 uropathogenic *E. coli strains isolated from* mid and last-stream urine of inpatients were clinically isolated from the urine samples of UTI patients in Henan Provincial People's Hospital and Nanyang First People's Hospital between May 2017 and November 2018. UPEC isolates were isolated from nonduplicate UTI patients and identified via a BD Phoenix 100 Automated Microbiology System (Becton Dickinson & Co., Franklin Lakes, NJ, USA). Furthermore, the presence of ESBLs-resistant phenotypes was identified using the BD Phoenix 100 system. *E. coli* ATCC 25922 was used as a quality control strain.

### 2.2. Antibacterial Assays (MIC)

The minimum inhibitory concentration (MIC) of fosfomycin was assessed using agar dilution according to CLSI guidelines with 25 *μ*g/ml glucose-6-phosphate [16, 17]. The concentration of fosfomycin on agar disks ranged from 0.05 to 512 *μ*g/ml. The MIC was defined as the lowest concentration of fosfomycin, with no visible growth of *E.coli.* Fosfomycin breakpoints were established according to the following CLSI standards: ≤64 *μ*g/ml as susceptible, 128 *μ*g/ml as intermediate, and ≥256 *μ*g/ml as resistant [17]. E. coli 25922 was used as a quality control strain.

### 2.3. PCR

The DNA genome of *E.coli* was extracted using the simple boiling method with a few modifications [18]. After DNA template isolation, VFs, including type 1 pili (*FimH*), P fimbriae (*papA, papC*), and iron acquisition (*IutA, ChuT*), were detected using polymerase chain reaction. The primer sequences for amplification are shown in (Supplementary Table 1) [19–21]. PCR was performed in a 50 *μ*l reaction system containing 25 *μ*l 2× Mix (Taq polymerase, dNTPs), 1.0 *μ*l DNA template, and 2 *μ*l of each primer (10 *μ*M). The procedure for gene amplification cycles was as follows: denaturation at 94°C for 5 min, followed by 55°C for 30 sec and 30 cycles of 72°C for 1 min, with a final extension of 5 min at 72°C. The PCR products were detected via agarose gel electrophoresis in 1% Tris-EDTA buffer. The gels were visualized and photographed with ultraviolet light. The results are shown as presence (+) or absence (-) of genes.

### 2.4. In Vivo Experiment

#### 2.4.1. Mouse Inoculations and Treatment

Animal studies were approved by the Experimental Animal Administration and Ethics Committee (Code: 2017-B150) of the Experimental Animal Center of Zhengzhou University (Zhengzhou, Henan, China). After culturing for 24 h, the *E. coli* strains from UTI patients were washed and resuspended in PBS at a concentration of 1 × 10^9^ CFUs/ml. The protocol for pyelonephritis induction was transurethral inoculation of bacteria, as previously described with slight modifications [22, 23]. Thirty BALB/c mice (purchased from the Henan Province Laboratory Animal Center, China) were divided into the following five groups (*n* = 6/group): healthy control, APN vehicle, 200 mg/kg fosfomycin, 400 mg/kg fosfomycin, and 800 mg/kg fosfomycin. The healthy control and APN vehicle groups received PBS, and the other three groups received a low (200 mg/kg), middle (400 mg/kg), or high dose (800 mg/kg) of fosfomycin via tail vein injection. After anaesthetization with isoflurane, the mice were catheterized with soft sterile polyethylene and transurethrally injected with 1 × 10^9^ CFUs/ml in 50 *μ*l into the urinary bladder. Immediately after the injection, the urethra was ligated with a thin thread and released after two hours. Mice were injected with PBS or fosfomycin 24 h after the establishment of the model. The mice were sacrificed for further renal histopathology evaluation and bacterial load analyses 24 h postinjection.

#### 2.4.2. Measurement of the Bacterial Load and Kidney Index

The kidney, bladder, and urine bacterial load of mice in different groups were measured by the bacterial plate count as previously described [10, 24]. The urine of mice was obtained by gently squeezing the lower abdomen. Subsequently, the bladder and two kidneys were aseptically removed. The kidney index was calculated according to the following equation [25]:

(1) $$\begin{array}{c} \text{Kidney index}=\frac{\text{kidney weight}\left(\text{mg}\right)}{\text{Bodyweight of the mice on the day of sacrifice }\left(\text{g}\right)}. \end{array}$$

Each kidney was cut longitudinally into 2 halves. One-half of the kidney was fixed in 10% formaldehyde and prepared for histopathological examination. The bladder and the other half of the left kidney were homogenized for bacterial load analyses. A serial dilution of homogenates was plated on the culture dish and cultured for 24 h at 37°C. The bacterial CFUs on the plates were counted and the results are expressed as CFUs per gram of kidney/bladder or milliliter of urine.

#### 2.4.3. Histopathological Examination

The other half of the left kidney was fixed in 10% formalin for 24 hours and embedded in paraffin. Paraffin sections (4 *μ*m) were then dehydrated with 30-100% ethanol and stained with hematoxylin and eosin (H&E). The histopathology (tissue destruction, cellular infiltration, and bacterial patchiness) of each kidney was analyzed with K-Viewer software (KFBIO Technology for Health Co., Ltd., China). The histopathological examination was performed by an experienced urinary tract pathologist.

### 2.5. Statistical Analysis

The chi-square test and Fisher's test were used to compare the VFs and ESBLs in UPEC isolates for statistical analysis. One-way analysis of variance was used for comparisons between multiple groups, and the results are expressed as the mean ± SD. The Mann–Whitney *U* test was used to compare the number of CFUs in the urine, bladder, and kidneys of different groups. The data were analyzed with SPSS 22.0 software (IBM Corporation, Stomer, NY, USA), and *p* < 0.05 was considered statistically significant.

## 3. Results

### 3.1. Characteristics of UPEC Resistance

A total of 22 UPEC strains were isolated from UTI patients in this study. The MIC of fosfomycin in the UPEC strain was 4 *μ*g/ml. The MIC values of clinical UPEC isolates may vary based on the geographic area and time. Twelve strains of the 22 isolated strains (12/22, 54.5%) showed an ESBL phenotype.

### 3.2. Distribution of Virulence Genes and ESBLs

The distribution of virulence genes in 22 UPEC strains and the quality control strain *E. coli* 25922 and the ESBLs phenotype are shown in Table 1. Among the 22 UPEC isolates, *FimH* was the most prevalent virulence gene factor (*n* = 22, 100%), followed by the iron acquisition-associated factors *ChuT* (*n* = 17, 77.3%) and *IutA* (*n* = 11, 50%), which are responsible for obtaining iron. Furthermore, the detection rate of the VFs P fimbria *papA* gene was *n* = 10, 45%, and that of *papC* was *n* = 11, 50%. All data are shown in Table 1.

### 3.3. Association between ESBLs and VFs

The prevalence of *FimH* among the phenotypical ESBLs-producing strains and non-ESBLs producing strains was 100%. The prevalence rates of the *papA* and *pap C* genes among ESBLs-positive strains were 42% and 50%, respectively. For the iron acquisition system, the prevalence rates of *chuT* and *IutA* were 75% and 33%, respectively. However, no significant relationship was found between the phenotype of ESBLs and VFs. All data are shown in Table 2.

### 3.4. Therapeutic Efficacy of Fosfomycin in the APN Model

#### 3.4.1. Comparison of the Kidney Index

**Figure 1:**
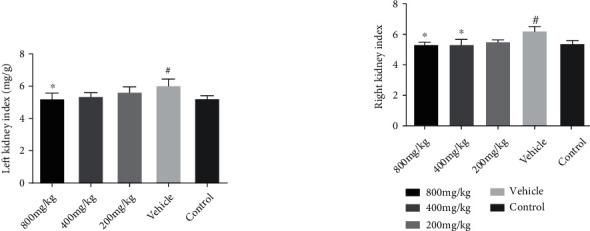
Changes in the kidney index among the low- (200 mg/kg), middle- (400 mg/kg), and high-dose (800 mg/kg) fosfomycin groups compared with the vehicle and control groups (APN model) (mean ± SD) (*n* = 6). ^#^Model (vehicle) group compared with the healthy control group, *p* < 0.05; ^∗^treatment group compared with the model (vehicle) group, *p* < 0.05.

The kidney index of APN mice in different groups is shown in Figure 1. Both the left and right kidneys showed a similar trend. The kidney index was significantly increased in the APN group compared to the healthy control group (*p* < 0.05). Compared to the model group, the high-dose of fosfomycin (800 mg/kg, equivalent to the dose of 6 g in human) group had a significantly lower kidney index (*p* < 0.05). The middle-dose fosfomycin (400 mg/kg) group had a significantly lower right kidney index than the model group (*p* < 0.05), but there was no statistical difference in the left kidney index (*p* > 0.05). The low-dose fosfomycin group showed a trend of a reduction in the kidney index, but the difference was not statistically significant (*p* > 0.05).

#### 3.4.2. Tissue Bacteriology

**Figure 2:**
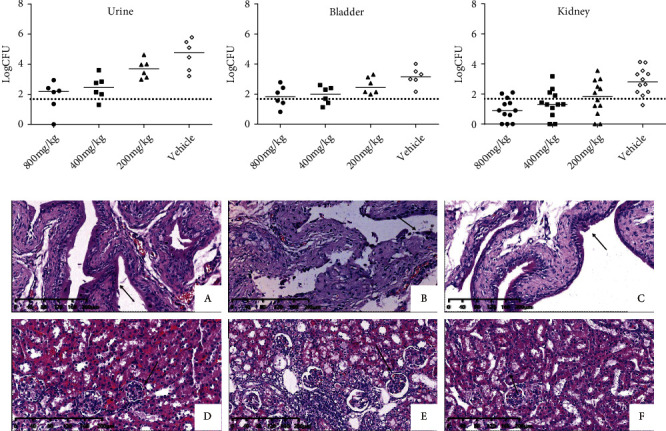
Effect of fosfomycin treatment on the UPEC-induced acute pyelonephritis model. Bacterial load (in log CFUs) in the urine (a), bladder (b), and kidney (c) of control and infected mice treated with fosfomycin. (d) Histopathological examination of bladder (A–C) and kidney (D‑F) sections. (A) Bladder, healthy control group; (B) bladder, APN group; (C) bladder, 800 mg/kg fosfomycin group; (D) kidney, healthy control group; (E) kidney, vehicle APN group; (F) kidney, 800 mg/kg fosfomycin group. Arrow in (A–C): bladder mucosa; arrow in (C–E): glomerular endothelial cells.

The bacterial load of the urine, bladder, and kidney of the APN group was significantly increased compared to that of the healthy control group (Figures 2(a)–2(c)). The middle- (400 mg/kg) and high- (800 mg/kg) dose fosfomycin groups exhibited a significant decline in bacterial counts in the urine and bladder compared to the vehicle group (*p* < 0.05) (Figures 2(a) and 2(b)). The results of the kidney bacterial load showed that all three doses (200 mg/kg, 400 mg/kg, and 800 mg/kg) of fosfomycin resulted in an increased clearance of bacteria compared to APN (*p* < 0.05) (Figures 2(a)–2(c)).

#### 3.4.3. Tissue Pathology

The results of the bladder and kidney histopathological examination are shown in Figure 2. The bladders of infected untreated mice showed severe bladder epithelial desquamation and neutrophil infiltration compared to those of healthy control mice (Figure 2(d, a, B)). Treatment with fosfomycin almost recovered bladder tissue with normal bladder epithelial cells and decreased inflammatory infiltration (Figure 2(d, C)). For kidney pathology, the infected mice showed severe neutrophil infiltration in the interstitial tissue, necrosis, vacuolization, and reduced glomerular endothelial cells in the renal tubule compared to the healthy control mice (Figure 2(d, E, F)). Examination of the kidney tissue of mice treated with fosfomycin showed reduced inflammatory infiltration cells and normalized glomeruli. All pathological changes among groups were corroborated with the bacterial loads of tissues.

## 4. Discussion

The high prevalence of multidrug-resistant (MDR) Gram-negative bacteria in UTI patients has posed a great threat to human health. As a normal flora of the human intestinal, the adherence of UPEC to uroepithelial cells is essential for the initiation of infection in UTI patients [26]. UPEC isolates are a genetically heterogeneous group of various virulence factors associated with the colonization and survival of bacteria in symptomatic or complicated UTIs [20, 26]. The specific virulence confers UPEC to adapt to the new location and causes a broad spectrum of diseases [26, 27]. As reported by previous studies, in *E. coli* strains that produce ESBLs, the extent of quinolone resistance is unexpectedly high. The relationship of virulence factors (*FimH*, *papA*, *papC*, *IutA*, and *ChuT*) remains determined.

In the present study, we isolated 22 *E coli.* strains from UTI patients analyzed the virulence factor and ESBL phenotype and evaluated the therapeutic effect of fosfomycin in a UPEC-induced acute pyelonephritis model. We observed that the detection rate of type I fimbriae *FimH* in 22 UPEC strains was 100%, and those of the P fimbria *papA* and *papC* genes were 45% (10/22) and 54% (12/22), respectively. These bacteria have strong pathogenicity, and more than 50% (12/22) of UPEC strains produced ESBLs, which was consistent with the data of Chinese CHINET bacterial resistance monitoring in 2015 and 2016. Type 1 fimbriae *FimH* and P fimbriae are involved in the occurrence and development of cystitis and pyelonephritis in UTI, respectively. The result of our study was also consistent with previous research showing that *FimH* was most frequent in isolates from a variety of UTIs [20]. Type 1 fimbriae *FimH* could induce the production of reactive oxygen species in leukocytes, which was related to the formation of pyelonephritis scars, and induced the expression of P fimbriae [28]. Furthermore, type 1 and P fimbria act in synergy to facilitate the colonization of *E coli*: P fimbriae enhances initial colonization of the tubular epithelium. In contrast, type 1 fimbriae mediate colonization of the center of the tubule via interbacterial binding and biofilm formation [29].

In addition, we found no correlation between the ESBLs and VFs. Previous studies have shown that the frequencies of the hlyD and iroN VFs are significantly different between ESBLs-producing and non-ESBLs-producing strains. [30]. This difference might be attributed to the source and number of selected strains. Although the frequency of VFs is closely associated with the pathogenicity of UTIs, the relationship between drug resistance and VFs has yet to be further studied. The production of ESBLs is one of the main reasons for bacteria to develop drug resistance, making them resistant not only to *β*-lactam antibiotics but also to fluoroquinolones, sulfonamides, aminoglycosides, and tetracycline antibiotics [15, 31]. However, ESBLs-producing *E coli.* show high sensitivity to fosfomycin.

For *in vivo* studies, E. coli CFT073, NU14, UTI89, and UPEC strains directly isolated from UTI patients are often used to establish UTI models [32–34]. We established an acute pyelonephritis model of ascending infection using *E coli*. isolated from UTI patients. Acute pyelonephritis is mainly caused by various microorganisms, among which the most important strain is *E. coli* [7]. The preferred treatment for ESBLs-producing Enterobacteriaceae invasive infection is c carbapenems [34]. However, with the long-term and unreasonable usage of antibiotics, drug resistance and side effects have gradually increased, which reduces its clinical therapeutic effect and makes a patient prone to relapse. The mechanisms of drug resistance mainly include producing serine carbapenemase and metallo *β*-lactamases (MBLs) or reducing the binding of porins to ESBLs or AmpC *β*-lactamases [35]. Therefore, it is important to find a replacement for carbapenems.

Fosfomycin is a broad-spectrum antibiotic that inhibits the formation of bacterial cell walls by competing with phosphoenolpyruvate transferase [11]. Bacterial resistance to fosfomycin is mainly attributed to chromosomes, and the cross-resistance between antibacterial agents such as beta-lactams and aminoglycosides is not significant [36, 37]. The ARESC study showed that 74.6% of patients were uropathogenic positive, and *E coli* accounted for 76.7% of isolates [38]. A multidose regimen of oral fosfomycin has been a potential strategy for complex urinary tract infections and prostatitis treatment. The oral formulation of fosfomycin trometamol has been approved for the treatment of clinical UTIs alone. Intravenous fosfomycin (ZTI-01) was recently developed for the treatment of complex UTIs and acute pyelonephritis in the United States. The phase 2/3 trial ZEUS results showed that fosfomycin exhibited a noninferiority effect compared to piperacillin-tazobactam in a microbiologically modified intention to treat population [7]. This offers us a new intravenous therapeutic option with a different mechanism of action for serious Gram-negative infection. In addition, the environment of urine (pH = 6.0) further enhances the antibacterial activity of fosfomycin [39].

From the results of our research, the intravenous administration of fosfomycin (800 mg/kg, equivalent to the dose of 6 g in the human body) reduced the bacterial load and significantly ameliorated the histopathological properties of the bladder and kidney. Our study enhances the evidence for the usage of fosfomycin for UTI patients. The clinical therapeutic effect of antimicrobial agents is closely related to the drug concentration at the site of infection and susceptibility to the pathogen. It is necessary to formulate an individualized treatment plan for UTI patients. Future research should be devoted to exploring the effect of fosfomycin on Gram-negative bacteria with different degrees of drug resistance in urinary tract infections. If necessary, the combination of fosfomycin with other antibacterial agents should be considered.

## 5. Conclusion

In conclusion, our study described the relationship between VFs and the ESBLs phenotype of *E coli.* isolates from UTI patients and evaluated the effect of fosfomycin in the APN model. The *E coli.* strains from UTI patients showed a high frequency of type 1 *FimH* gene (100) and the EBSL-producing phenotype (54.5%). However, there was no relationship between the VFs and the ESBL phenotype. Fosfomycin showed antibacterial activity towards clinically isolated *E coli*. strains *in vitro*. Furthermore, fosfomycin (800 mg/kg) showed an antibacterial therapeutic effect, including bacterial clearance and pathological improvement towards the kidney and bladder in the APN model. The above experimental results indicate that the intravenous administration of fosfomycin is effective for acute pyelonephritis caused by highly pathogenic and drug-resistant strains.

## Figures and Tables

**Table 1:** Distribution of virulence genes and ESBLs of 22 UPEC strains.

Strain no.	Virulence genes					ESBLs	Body site
	*FimH*	*papA*	*papC*	*ChuT*	*IutA*		
UP 1	+	+	+	+	-	+	Urine
UP 2	+	-	+	+	-	+	Urine
UP 3	+	-	+	+	-	+	Urine
UP 4	+	-	-	-	-	-	Urine
UP 5	+	+	+	+	+	+	Urine
UP 6	+	-	-	-	-	-	Urine
UP 7	+	+	+	+	+	-	Urine
UP 8	+	+	+	+	+	-	Urine
UP 9	+	-	-	-	-	+	Urine
UP 10	+	-	+	+	-	+	Urine
UP 11	+	+	+	+	+	-	Urine
UP 12	+	-	+	+	-	-	Urine
UP 13	+	-	-	+	+	-	Urine
UP 14	+	-	+	+	-	+	Urine
UP 15	+	+	-	+	+	-	Urine
UP 16	+	+	-	+	+	+	Urine
UP 17	+	-	-	-	-	+	Urine
UP 18	+	+	+	+	+	-	Urine
UP 19	+	-	-	+	+	-	Urine
UP 20	+	+	-	+	+	+	Urine
UP 21	+	+	-	+	+	+	Urine
UP 22	+	-	-	-	-	+	Urine
25922	+	+	+	+	+	-	

Values in the table represent the presence (+) or absence (-) of the genotype; UP: UPEC pattern.

**Table 2:** The correlation between ESBLs and virulence genes in 22 UPEC strains.

Virulence genes	Result	ESBLs^+^	Non-ESBLs^−^	Chi-square, *p* value
		n=12 (%)	*n* = 10 (%)	
*FimH*	+	12 (100%)	10 (100%)	NS
	_	0 (0)	0 (0)	
*papA*	+	5 (42%)	5 (50%)	NS
	-	7 (58%)	5 (50%)	
*papC*	+	6 (50%)	5 (50%)	NS
	-	6 (50%)	5 (50%)	
*ChuT*	+	9 (75%)	8 (80%)	NS
	-	3 (25%)	2 (80%)	
*IutA*	+	4 (33%)	7 (70%)	NS
	-	8 (67%)	3 (30%)	

NS, not significant.

## Data Availability

The data that support the findings of this study are available from the corresponding author upon reasonable request.

## Supplementary Materials

Supplementary Materials The sequence of primers used for the detection of virulence genes in E. coli isolates were listed in supplementary file.
