# Supplementary material for: Studies on Virulence and Extended-Spectrum β-Lactamase-Producing Uropathogenic Escherichia coli Isolates and Therapeutic Effect of Fosfomycin in Acute Pyelonephritis Mice
Source: Biomed Res Int. 2022 Jan 30;2022:8334153. doi: 10.1155/2022/8334153 (PMC8818418; doi:10.1155/2022/8334153)
Supplement: Supplementary Materials — The sequence of primers used for the detection of virulence genes in E. coli isolates were listed in supplementary file. [file 8334153.f1.docx]

**Studies on virulence and extended-spectrum β-lactamase producing uropathogenic Escherichia coli isolates, and therapeutic effect of fosfomycin in acute pyelonephritis mice**

**Supplentmary information:**

**Table 1.** The sequence of primers used for the detection of virulence genes in E. coli isolates

bp: base pairs; F: forward; R: reverse

| Gene type | virulence genes | Sequence (5'→ 3') | Size of PCR product (bp) | Annealing temperature (°C) |
| --- | --- | --- | --- | --- |
| Ⅰ pili | *FimH* | F:TGCAGAACGGATAAGCCGTGG  R:GCAGTCACCTGCCCTCCGGTA | 508 | 55 |
| P pili | *papA* | F:ATGGCAGTGGTGTTTTGGTG  R:CGTCCCACCATACGTGCTCTTC | 720 | 55 |
|  | *papC* | F:GTGGCAGTATGAGTAATGACCGTTA  R:ATATCCTTTCTGCAGGGATGCAATA | 200 | 55 |
| iron acquisition | *IutA* | F:GGCTGGACATCATGGGAACTGG  R:CGTCGGGAACGGGTAGAATCG | 301 | 55 |
|  | *ChuT* | F:GACGAACCAACGGTCAGGAT  R:TGCCGCCAGTACCAAAGACA | 279 | 55 |
